# Transcriptome analysis of polyploid giant cancer cells and their progeny reveals a functional role for p21 in polyploidization and depolyploidization

**DOI:** 10.1016/j.jbc.2024.107136

**Published:** 2024-03-04

**Authors:** Shai White-Gilbertson, Ping Lu, Ozge Saatci, Ozgur Sahin, Joe R. Delaney, Besim Ogretmen, Christina Voelkel-Johnson

**Affiliations:** 1 Department of Microbiology & Immunology, Medical University of South Carolina, Charleston, South Carolina, USA; 2 Department of Biochemistry and Molecular Biology, Medical University of South Carolina, Charleston, South Carolina, USA

**Keywords:** cancer biology, cell signaling, sphingolipid, ceramide, bioinformatics, stress response

## Abstract

Polyploid giant cancer cells (PGCC) are frequently detected in tumors and are increasingly recognized for their roles in chromosomal instability and associated genome evolution that leads to cancer recurrence. We previously reported that therapy stress promotes polyploidy, and that acid ceramidase plays a role in depolyploidization. In this study, we used an RNA-seq approach to gain a better understanding of the underlying transcriptomic changes that occur as cancer cells progress through polyploidization and depolyploidization. Our results revealed gene signatures that are associated with disease-free and/or overall survival in several cancers and identified the cell cycle inhibitor CDKN1A/p21 as the major hub in PGCC and early progeny. Increased expression of p21 in PGCC was limited to the cytoplasm. We previously demonstrated that the sphingolipid enzyme acid ceramidase is dispensable for polyploidization upon therapy stress but plays a crucial role in depolyploidization. The current study demonstrates that treatment of cells with ceramide is not sufficient for p53-independent induction of p21 and that knockdown of acid ceramidase, which hydrolyzes ceramide, does not interfere with upregulation of p21. In contrast, blocking the expression of p21 with UC2288 prevented the induction of acid ceramidase and inhibited both the formation of PGCC from parental cells as well as the generation of progeny from PGCC. Taken together, our data suggest that p21 functions upstream of acid ceramidase and plays an important role in polyploidization and depolyploidization.

Polyploidy, also known as whole genome duplication, has a protective effect in highly differentiated tissues such as the heart, liver, or muscle but is associated with poor prognosis in cancer due to increased genomic instability (1, 2). Stress within the tumor microenvironment or in response to therapy promotes polyploidy, particularly in cells with dysfunctional cell cycle regulation (3). Polyploid giant cancer cells (PGCC) are present in about one-third of tumors and while they present only a small fraction of the total cells, the danger lies in their ability to undergo depolyploidization with subsequent generation of progeny with self-renewal features (4, 5). The parallels between early embryonic development and PGCC suggest that dedifferentiation and reactivation of embryonic programs could be responsible for the plasticity and pluripotency observed in this subpopulation (5, 6, 7, 8). Because PGCC promote tumor heterogeneity, therapy resistance, and tumor recurrence, they have been described as the “evil roots of cancer” or the “keystone species” within the tumor microenvironment (9, 10, 11, 12). Despite detailed morphological description of these cells in human cancer specimen as well as studies in animal models and in cell culture, mechanisms involving polyploidization and depolyploidization in malignancy remain incompletely understood (13, 14, 15).

The expression of acid ceramidase, a lysosomal enzyme in the sphingolipid pathway that hydrolyses the pro-apoptotic sphingolipid ceramide to sphingosine, is elevated in several malignancies and has been identified as a therapeutic target in glioblastoma and in melanoma, where ablation of its activity interferes with cancer-initiating cells (16, 17). Pharmacological inhibition of acid ceramidase during radiation therapy led to durable cures in a preclinical model of prostate cancer that used the androgen-independent p53-null PPC1 model (18). In seeking to understand the underlying mechanism responsible for the durable cures, we discovered that acid ceramidase plays a crucial role in depolyploidization of PGCC that survive radiation stress (19). Formation of PGCC has been observed in multiple cancer models in response to various stresses and we have shown that restoration of normal p53 function in the PPC1 model significantly reduces polyploidization (13, 20). Neither acid ceramidase nor ceramide synthase 6 (CerS6), an enzyme that preferentially generates C_16_-ceramide, interfere with polyploidization but both enzymes regulate the ability of cells to undergo depolyploidization (20). RNA-seq analysis of PGCC treated with the acid ceramidase inhibitor LCL521 altered the expression of only four genes, including two involved in cholesterol metabolism (21). While treatment with LCL521 had no or little effect on mRNA expression levels in PPC1 and PPC1-PGCC respectively, we noted significant differences between PPC1 and PPC1-PGCC that were not associated with lipid metabolism (21). Therefore, the goal of this study was to gain a better understanding of the overall transcriptomic changes that occur as cancer cells progress through polyploidization and depolyploidization in response to stress. RNA-seq analysis identified the cell cycle inhibitor CDKN1A/p21 as a central hub in PGCC that persisted into early progeny. p21 is a multi-functional protein, widely known for its importance in cell cycle regulation and senescence (22). Data presented in this study suggest that p21 plays a functional role in polyploidization during the formation of PGCC as well as in depolyploidization during the generation of PGCC progeny, with signaling occurring upstream of acid ceramidase. Our data suggests that p21 plays a pivotal role in cancer recurrence.

## Results

### RNA sequencing shows significant differences as cancer cells transition through polyploidization and depolyploidization

**Figure 1:**
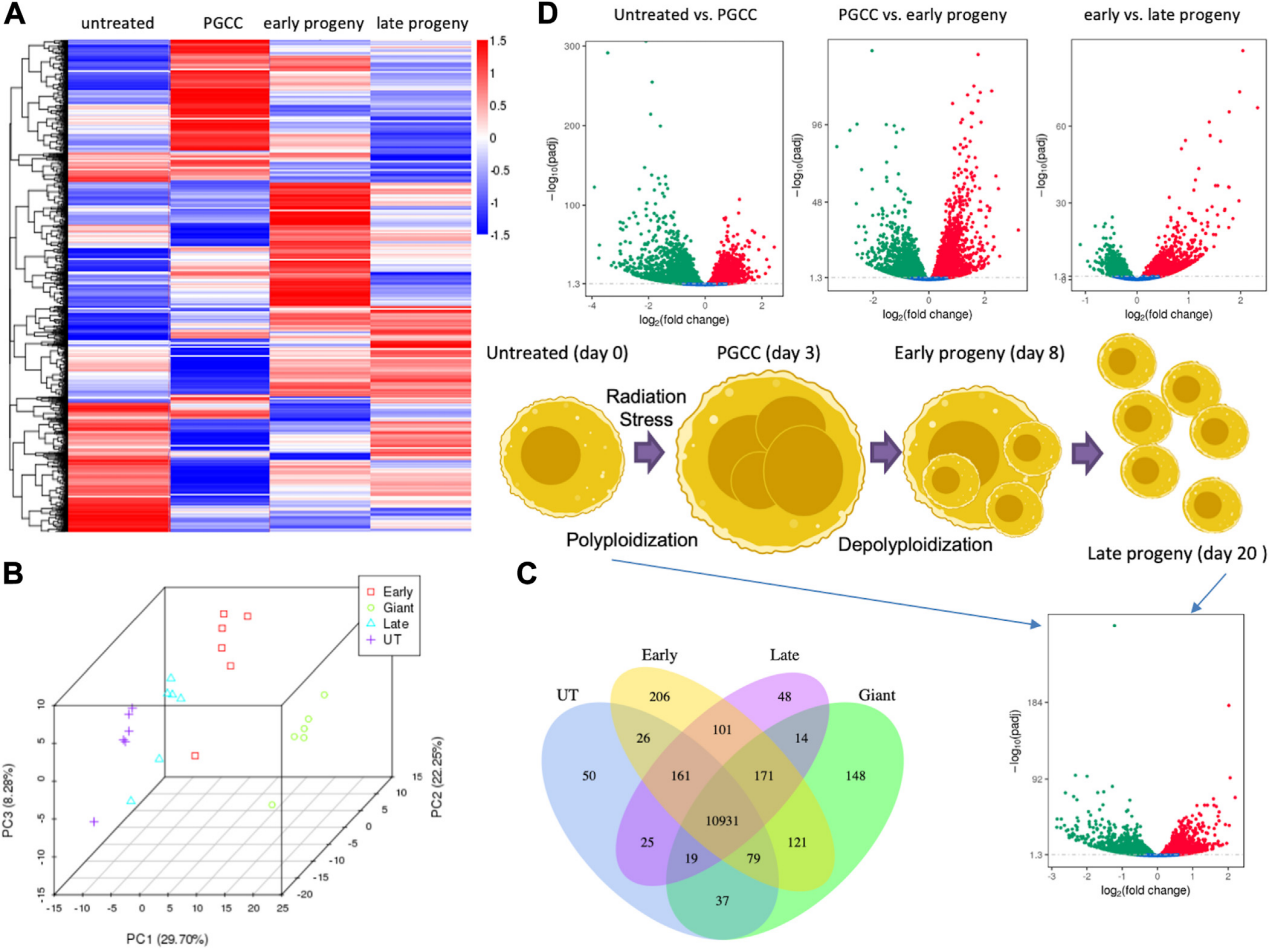
**RNA-seq analysis of PPC1 prostate cancer cells.** Four populations of PPC1 prostate cancer cells were analyzed from 6 independent RNA isolations. Differentially expressed genes are shown as heat map (*A*), Venn diagram (*B*) and volcano plots (*C*/*E*). Principal component analysis is shown in *D*.

The goal of this study was to elucidate the transcriptomic changes that occur as cancer cells progress through polyploidization and depolyploidization that allow for subsequent outgrowth. We initially used an RNA-seq approach to analyze gene expression across four populations in the human prostate cancer cell line PPC1. Comparisons were made between parental cells, PGCC obtained 3 days after radiation exposure, initial progeny that derive from the PGCC *via* asymmetric division on day 8 (“early”), and day 20 (“late”) (Fig. 1). Images of PGCC as well as videos of the depolyploidization process are found in our previous publications and we have also documented the increase in nuclear size in ovarian cancer models (19, 23, 24). Technical limitations of PGCC isolation include the lack of markers and the fragility of unfixed cells, which precludes recovery of viable cells after flow sorting (13, 23). Therefore, we routinely collect populations by a gentle “flow-through” filtration step using Pluri-Select filters, which results in a highly enriched population (21). Analysis of 48,162 genes revealed significant changes between groups (Fig. 1, *A* and *B*). Volcano plots indicate that 8436 genes were differentially expressed between untreated cells and PGCC (3969 genes upregulated and 4467 downregulated), 7680 genes were differentially expressed between PGCC and early progeny (3852 upregulated, 3828 downregulated), and 4143 genes were differentially expressed between early and late progeny (2025 upregulated, 2118 downregulated) (Fig. 1*C*). Principal component analysis demonstrates that the greatest differences are detected between untreated cells and PGCC, with profiles of early progeny shifting more towards untreated cells (Fig. 1*D*). While late progeny cells most closely approximate the untreated population, they retain significant differences with 2545 genes upregulated and 2667 downregulated compared to cancer cells prior to stress exposure (Fig. 1*E*).

Gene Ontology (GO) analysis of biological processes shows that genes involved in cell cycle checkpoint (GO: 0000075), DNA replication (GO: 0006260), and regulation of (mitotic) cell cycle phase transition (GO: 1901987/1901990) are altered throughout polyploidization and depolyploidization (untreated *versus* PGCC, PGCC *versus* early progeny, and early *versus* late progeny). Regulation of chromosome organization (GO: 0033044) and G2/M transition of (mitotic) cell cycle (GO: 0044839/0000086) differ between untreated cells and PGCC, whereas G1/S transition of (mitotic) cell cycle differs between early and late progeny (GO: 0044843/0000082). These results are consistent with G2/M arrest in PGCC and mitosis resuming in late progeny. Proteasomal protein (ubiquitin-dependent) catabolic processes (GO: 0010498;0043161) and response to endoplasmic reticulum stress (GO: 0034976) are differentially affected between untreated cells and PGCC as well as between PGCC and early progeny, which suggests that several processes altered in PGCC are maintained in the early progeny population. Biological processes that were uniquely different between PGCC and early progeny included regulation of serine/threonine kinase activity (GO: 0071900), intrinsic apoptotic signaling pathway in response to endoplasmic reticulum stress (GO: 0070059) and positive regulation of catabolic processes (GO: 0009896). As cells progress from early to late progeny biological processes involving (nuclear) chromosome segregation (GO: 0098813/0007059), mitotic nuclear division (GO: 0140014), and sister chromatid segregation (GO: 0000819) were differentially affected, which is consistent with re-entering the cell cycle. The full dataset is available from the Gene Expression Omnibus (GEO) database repository under accession GSE196453.

KEGG analysis showed enrichment of genes involved in cell cycle regulation, protein processing in the endoplasmic reticulum, pathways in cancer, p53 signaling as well as progesterone-mediated oocyte maturation throughout polyploidization and depolyploidization (untreated vs. PGCC, PGCC *versus* early progeny, and early *versus* late progeny). Adherens junctions, apoptosis, the insulin signaling pathway, glycerophospholipid, and sphingolipid metabolism emerged as significantly different when untreated cells were compared to PGCC. Differences in aminoacyl-tRNA biosynthesis, axon guidance, gap junction, and metabolic pathways were detected in PGCC and maintained in early progeny. Expression of genes associated with the MAPK and neurotrophin signaling pathways, endocytosis, spliceosome, and degradation of amino acids lysine, valine, leucine, and isoleucine differed between PGCC and early progeny. Differences in gene expression between early and late progeny included DNA replication, base excision repair, RNA transport, lysosome, oocyte meiosis, the one carbon pool by folate and amino acid metabolism (gly/ser/thr/ala/asp/glu metabolism and lysine degradation). The top three most significant differences that were retained among biological processes in late progeny compared to parental cancer cells were related to the Wnt signaling pathway (GO: 01987738/cell-cell signaling by Wnt *p* = 2.2 × 10^−12^; GO: 0016005/Wnt signaling pathway *p* = 2.83 × 10^−12^; GO: 0060070/canonical Wnt signaling pathway *p* = 3.43 × 10^−10^) and in KEGG analysis “pathways in cancer” emerged as the most significant difference (UT-PGCC *p* = 0.00011946, PGCC-early *p* = 5.87E-06 and early-late *p* = 0.00031377).

### Gene signatures are associated with cancer outcomes

To identify the most significant changes, we compared differentially expressed genes with an adjusted *p*-value of 0.01 and log_2_FC = 1.5 between groups and identified 373 genes that constituted a “PGCC signature”. Pathways enriched within this PGCC signature included chromatin assembly, metabolism, inflammation, growth factor signaling, and extracellular matrix regulation-related pathways. Differences in early and late progeny included immune activity, cell migration, cytokine production, and transcriptional regulation-related pathways. To determine the translational relevance of these findings, we stratified numerous datasets by the PGCC signature and found that it significantly predicted disease-free or overall survival in multiple types of cancers (Fig. S1).

Comparison of the PGCC signature from PPC1 prostate cancer cells with a panel of genes identified as stemness markers and radiation resistance in Du145 prostate cancer cells revealed an overlap of 16 and 31 genes, respectively, which supports earlier reports that PGCC exhibit stemness features and increased therapy resistance (Tables S1 and S2) (7, 8). Expression of several genes linked to the senescence-associated secretory phenotype (SASP), including IL-1β, IL-8, GLB1/β-galactosidase, TP53/p53, and CDKN1A/p21 was also significantly increased in PGCC relative to parental cancer cells (Fig. S2*A*). Treatment of PGCC with the senolytic drug Navitoclax reduced generation of progeny as evidenced by lack of colony formation and promoted PARP cleavage (Fig. S2, *B*–*D*). This is consistent with the drug’s mechanism of action and suggests that PGCC are functionally senescent (25).

**Figure 2:**
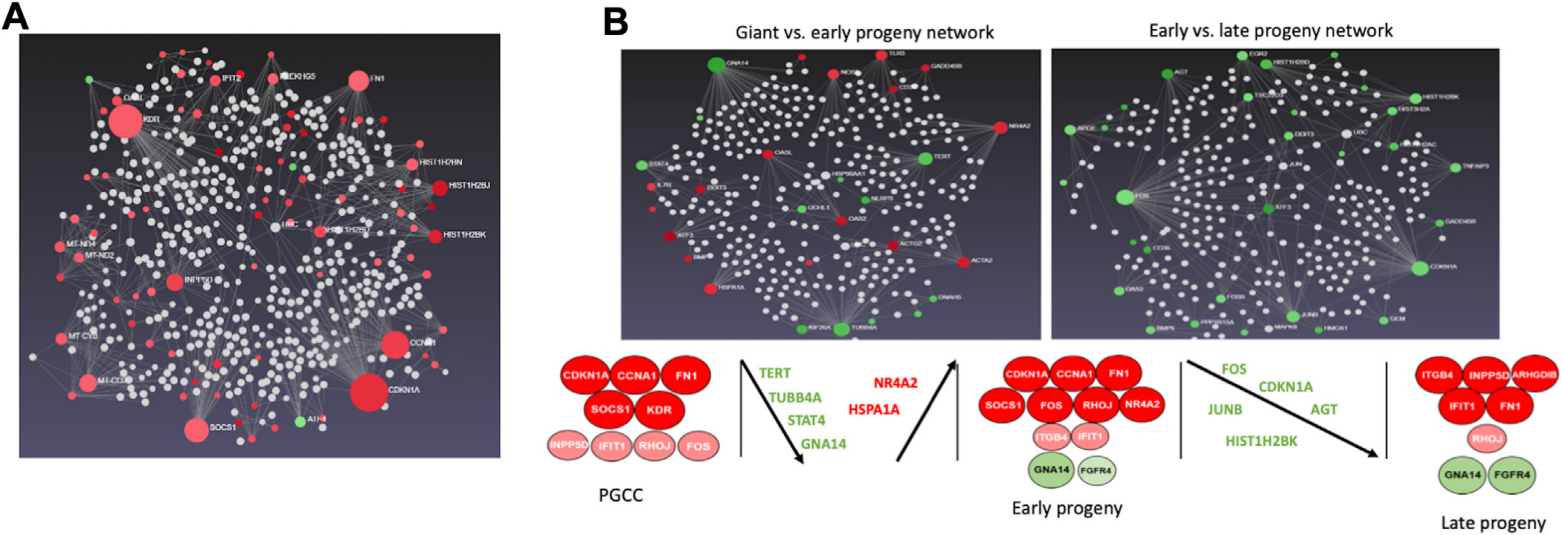
**Network Construction and Clinical Relevance of the gene expression changes upon radiation treatment.** The network Analyst database was used to generate the networks. The size of the nodes is based on their degree values, with a big size for large degree values. The color of nodes is proportional to their betweenness centrality values. *Red* colors are for upregulated genes and *green* are for downregulated genes. *White* nodes are additional proteins that are not found in the list. *A*, five genes with the highest degree and betweenness in PGCC are CDKN1A, CCNA1, FN1, KDR, and SOCS1. *B*, comparison of changes that occur as PGCC transitions to early and late progeny. (*A*) is also shown in Fig. S3 (*upper left panel*).

Network analysis identified the most important hubs of each cell state (Fig. S3). The most highly upregulated gene in PGCC was CDKN1A/p21, which together with CCNA1/cyclin A, SOCS1, KDR/VEGFR2, and FN1/fibronectin constituted a 5-gene core signature (Fig. 2*A*). Differences between PGCC and early progeny show decreases in GNA14, TERT and TUBB4A, but comparison between the early progeny population and untreated cells indicates that only the G-protein subunit GNA14 is an important hub in early progeny (Fig. 2*B*). Upon transition from early to late progeny, expression of many genes, including FOS and CDKN1A that were expressed in the previous cell state(s), decrease. While CDKN1A/p21, CCNA1/cyclin A, and SOCS1 remained major hubs in the early progeny, only FN1/fibronectin persisted as a major hub into late progeny (Figs. 2*B* and S3).

**Figure 3:**
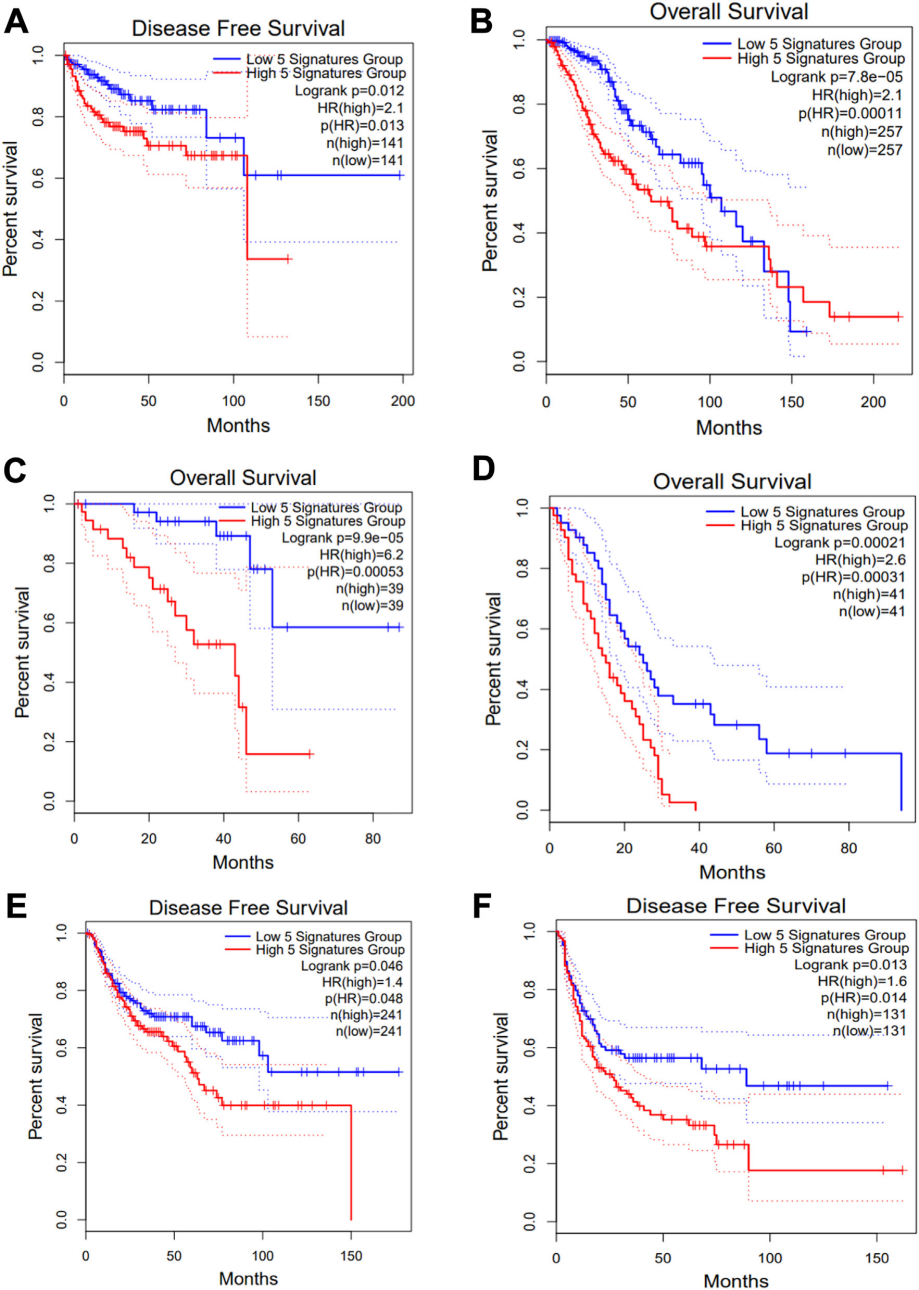
**Survival analysis in cancer differentiated by the 5-gene PGCC core signature.***A*, kidney renal papillary cell carcinoma, (*B*) low-grade glioma, (*C*) uveal melanoma, (*D*) mesothelioma, (*E*) lung squamous cell carcinoma, and (*F*) sarcoma. Survival curves were generated using the Kaplan-Meier method, and significance between groups was calculated by the Log-rank test.

Application of the 5-gene PGCC core signature was similarly predictive of outcomes in the same datasets used for the broad PGCC signature (Fig. 3, *A*–*C*) as well as in additional datasets in which application of the broad PGCC signature failed to reach significance (Fig. 3, *D*–*F*). One notable exception was prostate cancer in which neither of the PGCC signatures but rather the 5-gene core signature of late progeny (FN1, INPP5D, ITGB4, ARHGDIB, IFIT1) was significantly predictive of outcomes (Fig. S4).

### Elevated p21 expression in PGCC is detected in the cytoplasm

**Figure 4:**
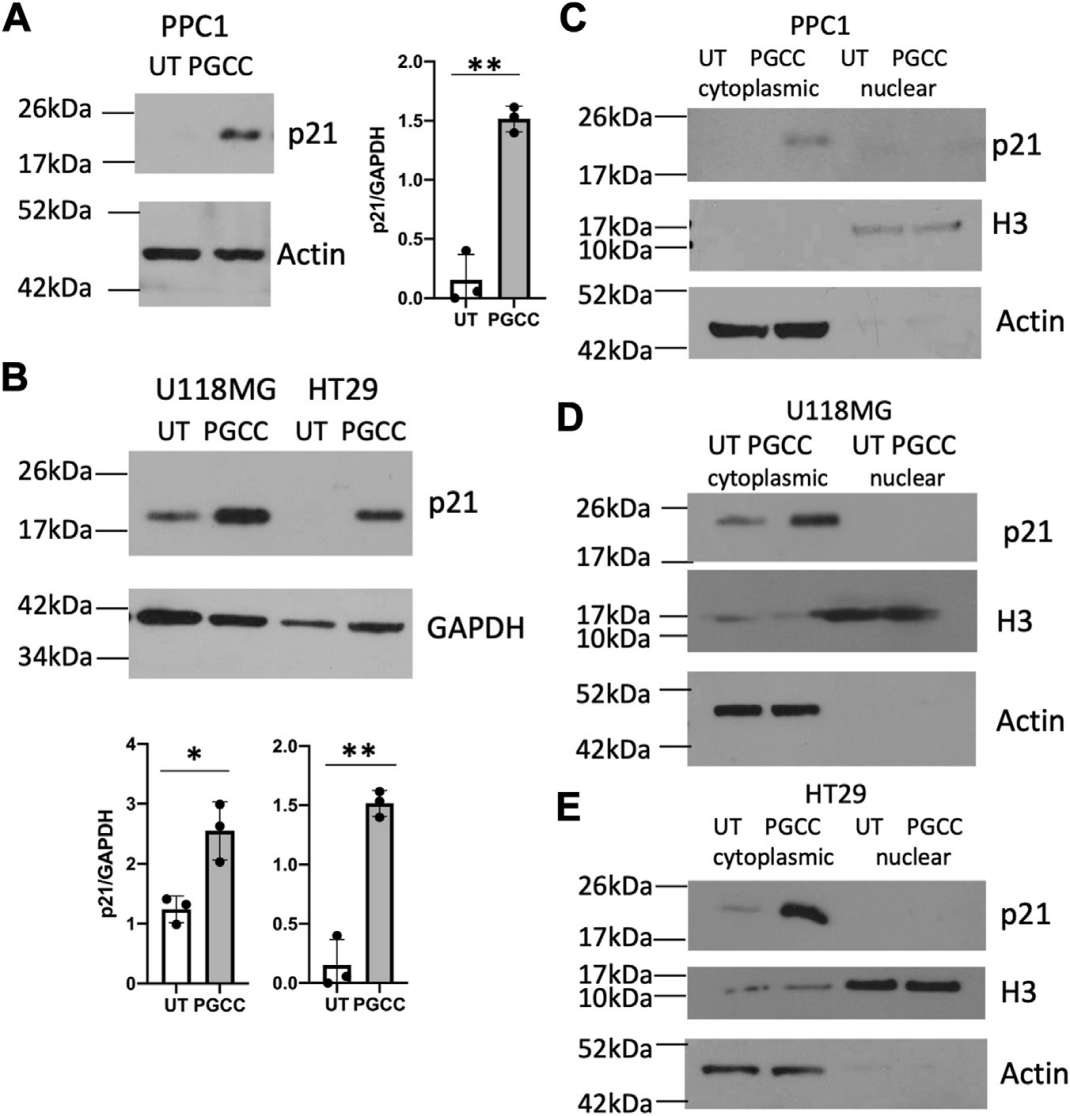
**Expression of p21 in PGCC.** Western blot analysis was performed to verify the upregulation of p21 mRNA at the protein level in PGCC compared to untreated cancer cells (UT) (*A* and *B*). Three independently performed experiments were analyzed for expression of p21 relative to GAPDH. ∗*p* < 0.05, ∗∗ *p* < 0.005. (*C*–*E*) Distribution of p21 in untreated cells and PGCC derived from PPC1, U118MG, and HT29 cells. Similar results were obtained in three independent experiments.

The cell cycle regulator p21 emerged as the major hub in PGCC, and therefore we further investigated its role in polyploidization and depolyploidization. We confirmed that the upregulation of p21 mRNA observed in the RNA-seq analysis corresponded to increased p21 protein expression in PGCC derived from PPC1 prostate cancer cells (Fig. 4*A*). As shown in Figure 4*B*, increased p21 expression was also detected in U118MG glioblastoma cells and HT29 colon cancer cells. p21 interacts with cyclins, cyclin-dependent kinases, or PCNA and is well-established for its role in maintenance of genome integrity at multiple points of the cell cycle (26, 27). Association of p21 with PCNA, a nuclear protein with DNA replication master regulator function, results in inhibition of DNA synthesis (26). However, phosphorylation by Akt on Thr145 and/or Ser146 facilitates the cytoplasmic location of p21, where it interacts with different binding partners, to promote apoptosis resistance, migration, and invasion (22, 28). To determine the subcellular localization of p21, we fractionated cells before (UT) and after stress that induced polyploidization (PGCC). Expression of p21 in nuclear fractions for which Histone H3 served as a marker was below levels of detection (Fig. 4, *C*–*E*). The cytosolic fraction of cells for which actin served as a marker, had no or low levels of p21 in untreated cells but showed increased expression of p21 expression in PGCC (Fig. 4, *C*–*E*).

### Upregulation of p21 is not dependent on ceramide or acid ceramidase

**Figure 5:**
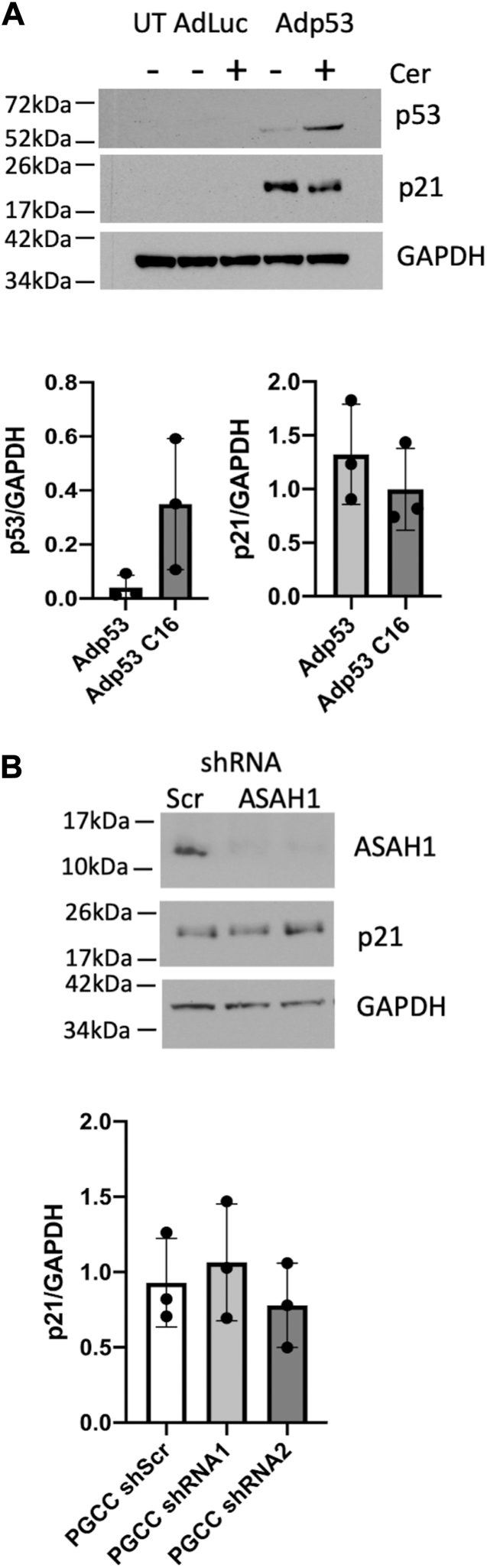
**Regulation of p21 in the context of ceramide and acid ceramidase in PPC1 cells.***A*, Western blot analysis of cells transduced with 1MOI of AdLuc or Adp53 followed by treatment with 5 μM exogenous pyridinium C_16_-ceramide. Expression of p53 and p21 (when detected) was quantified relative to GAPDH in three independently performed experiments. *B*, Western blot analysis of acid ceramidase and p21 in cells stably expressing an inducible control shRNA (scr, lane 1) and two shRNAs that target acid ceramidase (lanes 2 and 3). Following induction of the shRNA with doxycycline, cells were irradiated with 8 Gy and protein expression was analyzed on day 3. Three independently performed experiments were analyzed for expression of p21 relative to GAPDH. Differences were not significant.

Next, we focused on the interconnection of p21 and sphingolipid metabolism. Sphingolipids impact the expression of numerous genes (29). The key sphingolipid metabolite ceramide, which increases in response to many stressors, has been reported to mediate growth arrest in a p21-dependent manner, promote G1 arrest in hepatoma cells, and to drive p21-dependent G2 arrest in rhabdomyosarcoma cells (30, 31, 32). PPC1 cells are related to PC3 cells and express only one mutated copy of p53 that is not translated into protein due to an in-frame stop codon. Since induction of p21 can occur in a p53-dependent or p53-independent manner, we transduced PPC1 cells with wildtype p53 (22). The adenoviral vectors Adp53 and AdLuc were used at 1 copy/cell (33). After 24 h, a water-soluble pyridinium C_16_-ceramide, previously shown to stabilize p53, was added for an additional 24 h (34, 35). Expression of p21 was detected only in PPC1 cells transduced with Adp53 and while pyridinium C_16_-ceramide stabilized p53 protein, p21 expression was not further increased compared to Adp53 alone (Fig. 5*A*). We also tested if knockdown of acid ceramidase, which hydrolyses ceramide, affects p21 expression in response to radiation stress but did not observe any differences (Fig. 5*B*) (19). These data suggest that ceramide is not sufficient to drive p53-independent induction of p21 and that upregulation of p21 occurs either independently from or upstream of acid ceramidase.

### p21 is required for polyploidization and depolyploidization

**Figure 6:**
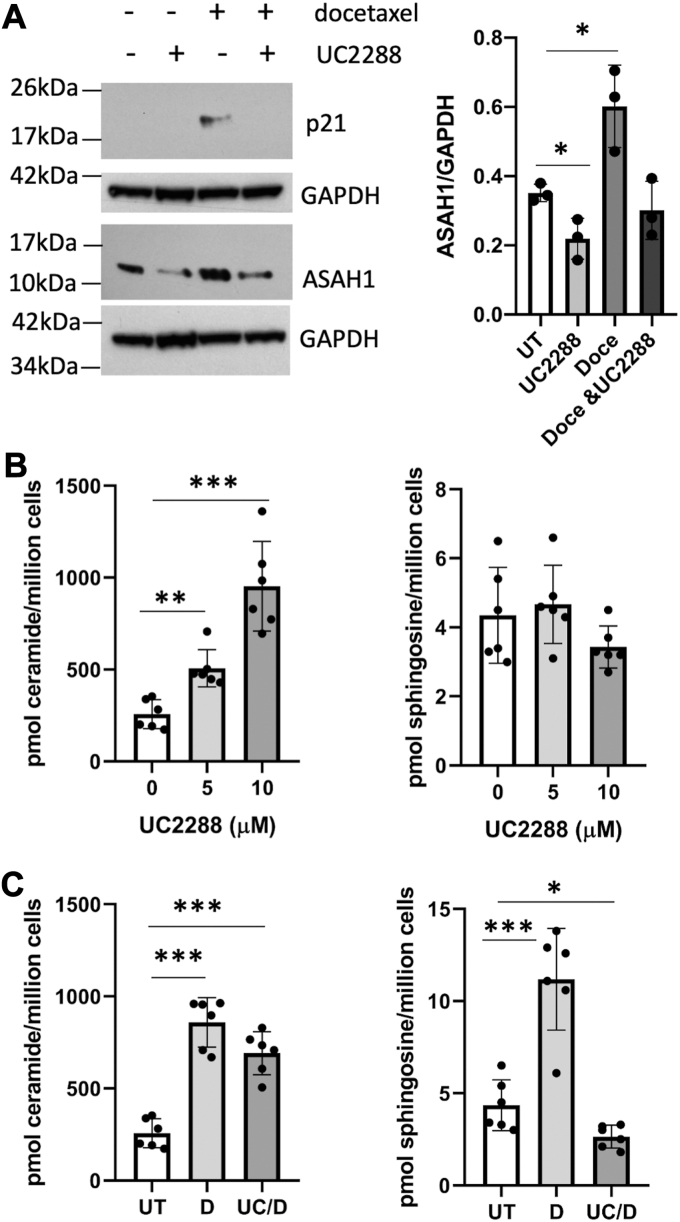
**The effect of UC2288 on protein expression and sphingolipids.***A*, Western blot analysis of PPC1 cells treated with 10 μM UC2288 and 5 nM docetaxel. Three independently performed experiments were analyzed for ASAH1 expression relative to GAPDH. Two-way ANOVA indicated differences between groups and was followed up with an unpaired, 2-tailed *t* test to evaluate differences between groups; ∗ *p* < 0.05. *B*, the effect of UC2288 on ceramide and sphingosine. *C*, the effect on 5 nM docetaxel alone or in combination with 10 μM UC2288 on ceramide and sphingosine. Lipid data are from two independent experiments performed in triplicate. Data shown in (*B*) and (*C*) were analyzed by an unpaired 2-tailed *t* test; ∗ *p* < 0.05; ∗∗ *p* < 0.005, ∗∗∗ *p* < 0.0005.

To determine whether p21 is merely a marker of PGCC or whether it played a functional role in this cell population, we performed several sets of experiments using the p21 inhibitor UC2288. UC2288, which is structurally similar to sorafenib but does not inhibit Raf or Akt, attenuates p21 protein levels through transcriptional regulation and functions independent of p53 (36). To determine the effect of UC2288 on p21 protein expression, we treated PPC1 cells with UC2288 alone or in combination with docetaxel stress. The increase in p21 expression in stressed cells was efficiently prevented by UC2288 and interestingly, the inhibitor also interfered with acid ceramidase expression (Fig. 6*A*). Treatment with UC2288 alone significantly increased total ceramide but did not alter sphingosine levels (Fig. 6*B*). In docetaxel stressed cells, UC2288 did not affect ceramide levels but prevented the increase in sphingosine (Fig. 6*C*). In fact, sphingosine levels in cells treated with UC2288 and docetaxel were significantly lower compared to untreated controls, which is consistent with protein expression analysis shown in Fig. 6*A*.

**Figure 7:**
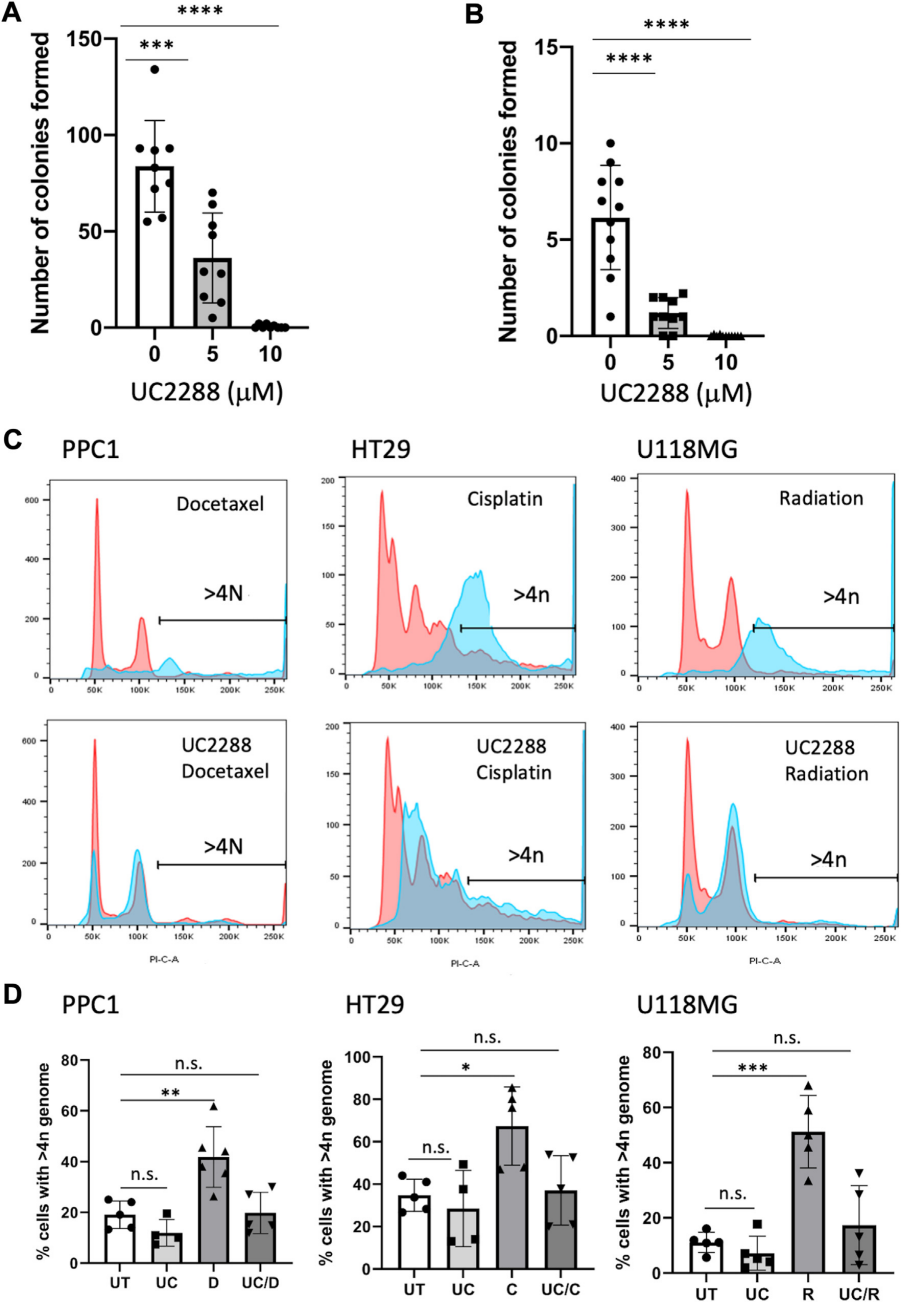
**The effect of UC2288 on depolyploidization and polyploidization.***A* and *B*, colony formation assay of PGCC derived from PPC1 (*A*) or U118MG cells (*B*). Data are from three independent experiments with 2 to 4 technical replicates. *C* and *D*, analysis of DNA content following a 3-days stress exposure. *C*, representative flow cytometry traces. *D*, quantification of flow cytometry data from three independent experiments. Two-way Anova indicated differences between groups and was followed up with a unpaired, 2-tailed *t* test to evaluate differences between groups; ∗ *p* < 0.05; ∗∗ *p* < 0.005, ∗∗∗ *p* < 0.0005.

To evaluate the role of p21 in depolyploidization, we treated PPC1-derived PGCC with UC2288 and found that colony formation was reduced significantly at 5 μM and completely prevented at 10 μM (Fig. 7*A*). Similar results were obtained in U118MG glioblastoma cells (Fig. 7*B*). To evaluate the role of p21 in polyploidization, we co-treated cells with therapeutic stressors in the absence or presence of UC2288 followed by flow cytometry analysis for multiple genomes on day 3. Results indicate that unlike ablation of acid ceramidase, UC2288 prevented polyploidy (Fig. 7, *C* and *D*) (19). Taken together, these results suggest that p21 functions upstream of acid ceramidase and is important for both polyploidization and depolyploidization of cancer cells as part of the stress response.

## Discussion

Polyploidy also known as whole genome duplication is an important event during development, tissue repair, and regeneration but is detrimental in malignancy. Genome doubling in cells with defective p53 results in a predictive pattern of genomic instability, which is permissive for tumor heterogeneity and therapy escape (37). Several independent groups have shown that polyploid tumor cells (PGCC) can generate progeny with self-renewal features that repopulate the tumor, which led to increased recognition of this subpopulation of cells as important drug targets (1, 9, 38, 39). The goal of this study was to gain an understanding of the overall transcriptomic changes that occur as cancer cells progress to PGCC following exposure to therapy stress, form initial progeny by asymmetric division, and transition to late progeny that resume proliferation. RNA-seq analysis revealed significant changes and provided molecular evidence that suggest the initial PGCC progeny are generated by non-mitotic pathways such as budding or bursting, which is consistent with previous microscopic observations (4, 6, 40). Gene expression profiles also suggest that PGCC and early progeny may evade and/or negatively influence the anti-tumor immune response. Immunosurveillance mechanisms against polyploid cells exist and evidence that PGCC formation and tumor recurrence can occur *in vivo* has been shown in a syngeneic rat model following cisplatin therapy (41, 42, 43). Future studies are needed to specifically evaluate how PGCC impact or evade anti-tumor immune responses.

We identified a 5-gene PGCC core signature that included CDKN1A/p21, CCNA1/cyclin A1, SOCS1/suppressor of cytokine signaling 1, KDR/Vascular Endothelial Growth Factor Receptor 2, and FN1/fibronectin, that was predictive of outcomes in several malignancies. While we focused our investigation on p21, the other four genes are also recognized for their roles in cancer. For example, CCNA1/cyclin A1 has recently been suggested as a potential diagnostic marker in papillary thyroid carcinoma and is a chemoresistance-associated biomarker in ovarian cancer (44, 45). In the latter study, ectopic expression of cyclin A1 decreased proliferation and increased senescence-associated β-galactosidase activity, which is consistent with increased senescence markers detected in PGCC. CCNA1 plays a role in the control of the germline meiotic cycle and increased expression in PGCC may be reflective of the dedifferentiation process triggered in malignant polyploidy (6). SOCS1 has been primarily investigated in immune cells, where it functions as a potent inhibitor of inflammation through negative regulation of cytokine-activated JAK-STAT pathways but is emerging with important functions in cancer where it antagonizes cell growth, suggesting that SOCS1 expression in PGCC may contribute to their non-proliferative state (46, 47). KDR is involved in the regulation of endothelial proliferation, survival, migration, tubular morphogenesis, and sprouting, which is consistent with an increase in the axon guidance pathway in PGCC. In the context of PGCC, KDR may function to promote depolyploidization through non-mitotic processes, which involves the formation of cytoplasmic extensions that visually resemble the axon formation of neurons (48). Fibronectin is a large glycoprotein dimer that is expressed in many human cancers where it facilitates a pro-tumor microenvironment (49). FN1 was the only gene among the 5-gene PGCC core signature that remained elevated in early and late progeny. Cellular fibronectin enables several hallmarks of cancer, including avoiding immune destruction, which may be important for the survival of PGCC, but also for promoting invasion and metastasis, sustaining proliferation, modulating cellular energetics, and inducing angiogenesis, all of which are likely important in depolyploidization of PGCC (49, 50).

Interestingly, while the RNA-seq analysis was performed in a prostate cancer cell line, the PGCC signature was not predictive of the outcome in this malignancy. Instead, we found that the 5-gene core signature of late progeny cells (FN1, ITGB4/integrin β4, INPP5D, ARHGDIB, and IFIT1) was associated with poor survival in two different datasets. While none of these genes by themselves are prognostic in prostate cancer, several have been associated with pro-tumor functions in other cancers (proteinaltlas.org). For example, ITGB4^+^ cancer stem cells have an intermediate epithelial/mesenchymal phenotype and elevated ITGB4 was associated with worse relapse-free survival in triple-negative breast cancer (TNBC) (51). Another study in TNBC shows that secretion of ITGB4 from cancer cells via exosomes triggers glycolysis in cancer-associated fibroblasts and the gene has also been reported as a novel prognostic factor in colon cancer (52, 53). Little is known about the remaining 3 genes that were identified in the 5-gene core signature in late progeny. ARHGDIB can mediate epithelial-mesenchymal transition through the downstream effector MMP-2 as high expression of this gene is indicative of a poor prognosis in breast cancer patients (54). IFIT1 belongs to a family of 4 genes that are stimulated by interferons and the current understanding of their role in cellular signaling is limited to innate immunity and the immune response to viruses. However, a prognostic significance in cancer has been suggested for IFITs and a novel role for IFIT1 and IFIT3 has been suggested in progression and metastasis in oral squamous cell carcinoma through interaction with annexin A2, which enhances recycling of the EGF receptor (55, 56). Future studies are needed to further determine the roles of these genes in prostate cancer.

The cell cycle inhibitory protein CDKN1A/p21 was selected as the focus for further investigation because it emerged as the most significantly changed gene in PGCC and persisted as a central hub in early progeny. We have shown that ceramide, primarily C_16_-ceramide, is increased in PGCC and that acid ceramidase is transcriptionally induced as described in detail by Cheng *et al*. (18, 19). Since the knockdown of acid ceramidase did not affect p21 expression, our data suggest that p21 functions independently from or upstream of the lysosomal enzyme. This is also supported by our previous study in which treatment with the acid ceramidase inhibitor LCL-521 had no effect on p21 mRNA upregulation in response to radiation (GSE195919) (21).

Treatment with UC2288, which prevented the expression of p21 in PGCC, reduced acid ceramidase expression, although an off-target effect on the enzyme, as observed with tamoxifen or carmofur, has not been ruled out (57, 58). In unstressed cells, UC2288 increases ceramide, but sphingosine is not significantly decreased. Therefore, the UC2288-mediated increase in ceramide in these cells is likely not due solely to the accumulation of acid ceramidase substrate and could occur through increased sphingomyelinase activity or *de novo* synthesis. In PGCC increased ceramide was detected near the cell surface and treatment with the acid ceramidase inhibitor LCL521 resulted in the accumulation of this pool (21). We hypothesize that increased ceramide in PGCC results from cleavage of sphingomyelin by acid or neutral sphingomyelinase in response to radiation or chemotherapy, respectively, and that acid ceramidase is necessary for the hydrolysis of this pool of ceramide (59, 60). Here we show that UC2288 reduces acid ceramidase expression below baseline and significantly decreases sphingosine in docetaxel treated cells. This presumably reflects the low levels of acid ceramidase expression and inability of UC2288 treated PGCC to hydrolyze ceramide that is liberated from sphingomyelin. Therefore, LCL521 and UC2288 may inhibit depolyploidization through a similar acid ceramidase-dependent mechanism.

Our data in PPC1 cells suggest p21 is induced in a p53-independent manner in PGCC, although mutant p53 may contribute to the higher levels detected in U118MG and HT29 cells. The p53-independent expression of p21 could be mediated by TGFβ, since mRNA is significantly increased in PGCC (*p* = 3.4917 × 10^−11^) (61). Regardless of p53 status, p21 was primarily detected in the cytoplasm of PGCC, where it may be retained by Akt-mediated phosphorylation on Thr145 and/or Ser146 (62). In trophoblastic giant cells Akt phosphorylates p21 and maintains the protein in the cytoplasm throughout G- and S-phases of endocycles, which protects cells from DNA damage induced apoptosis (63). Protection of apoptosis by cytoplasmic p21 occurs through interaction with binding partners such as caspases or ASK1 (22). The phosphorylation status of p21 in PGCC, identification of cytoplasmic interacting partners, and the consequences of binding partner interaction remain to be determined. The clinical relevance of cytoplasmic p21 is underscored by several studies. In gastric cancer multivariate regression analysis showed that low nuclear and high cytoplasmic levels of p21 were negative markers of overall survival (64). In malignant thymic epithelial tumors, cytoplasmic expression of p21 significantly correlated with decreased overall survival, progression-free survival, and metastasis-free survival (65). In head and neck squamous carcinoma, expression of p21 correlated with locoregional relapse and poor overall survival and interestingly patients with the worst overall survival were those with co-expression of p21 and Ki67, which was primarily detected after radiation therapy (66). The correlation between low nuclear/high cytoplasmic ratios of p21 and shorter overall survival may be due to therapy resistance (67, 68, 69, 70).

Chronic expression of p21 independent from p53 deregulates the replication licensing machinery by inhibition of the CRL4-CDT2 ubiquitin ligase and upregulation of replication licensing factors, including CDC6 (71). We found that CDC6 mRNA was significantly upregulated in PPC1-derived PGCC (P_adj_ = 1.35 × 10^−6^) and that Orc2, a licensing protein that remains bound to DNA replication origins throughout the cell cycle, was decreased (p_adj_ = 3.4591 × 10^−17^). Interestingly, a similar decrease in Orc2 mRNA was observed in PGCC derived from HEPA 1 to 6 or Hey cells (72, 73). During early embryonic development, Orc2 initially localizes to spindle poles and subsequently to the area between separating chromosomes (74). Orc2 depleted cells can still replicate but become critically dependent on CDC6 for survival and DNA replication (75). CDC6 has been identified as a factor that mediates radiation resistance through altering senescence, apoptosis, and epithelial-mesenchymal transition, all of which are altered in PGCC (76). Inhibiting expression of cytoplasmic p21 with UC2288 may prevent deregulation of the replication licensing machinery and therefore multiple genomes, characteristic of PGCC, do not accumulate.

**Figure 8:**
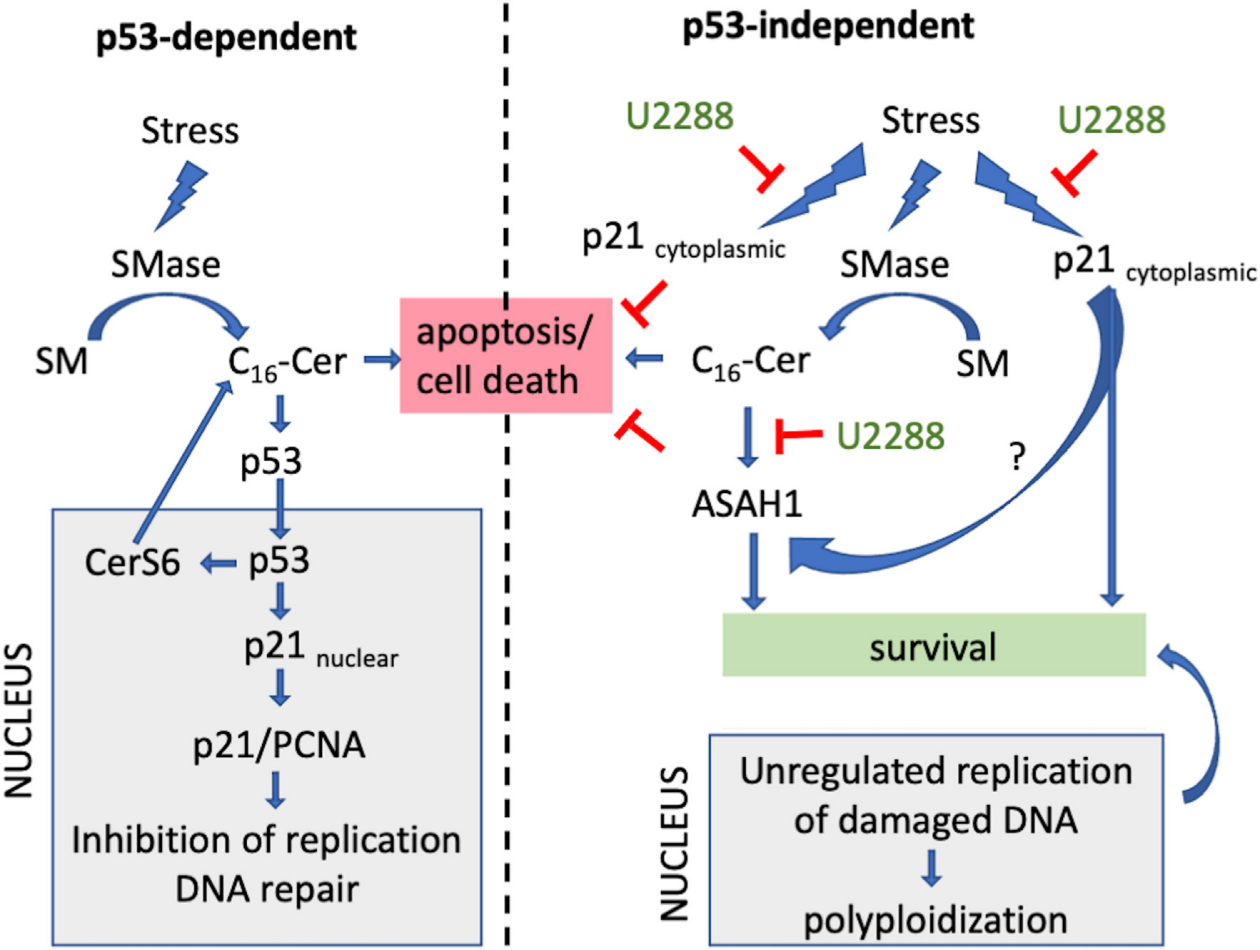
**Proposed model of p53-dependent and p53-independent signaling in response to stress.** Cell stress induces the activity of sphingomyelinases and preferentially generates C_16_-ceramide. The *left panel* shows responses of cells with normal p53 function. C_16_-ceramide stabilizes p53 and downstream targets CerS6 and p21 are induced. CerS6 generates more C_16_-ceramide and creates a positive feedback loop to maintain p53 stability. In the nucleus, p21 associates with PCNA to inhibit replication and allows for p53-mediated repair of DNA damage. The *right panel* shows p53-independent induction of p21. p21 localizes to the cytoplasm where it interacts with binding partners that suppress apoptosis/cell death. Cytoplasmic p21 deregulates replication licensing factors. Damaged DNA is not repaired by p53 and continued replication results in polyploidy. In parallel, ceramide transcriptionally activates acid ceramidase expression (ASAH1) to hydrolyze ceramide and promote cell survival. Polyploid cells that contain multiple copies of damaged DNA are capable of undergoing depolyploidization when ceramide levels are reduced. UC2288 interferes with p21 expression and polyploidy. UC2288 also reduces acid ceramidase expression and thereby prevents depolyploidization in PGCC. UC2288 may affect acid ceramidase directly or through an off-target effect.

Our current understanding of how sphingolipid metabolism and p21 affect polyploidization and depolyploidization is summarized in Figure 8. We propose that sphingomyelinases are activated in response to stress to generate ceramide from the most abundant species, which in cells we have tested is C_16_-SM (20). In normal cells, C_16_-ceramide liberated from sphingomyelin stabilizes p53 and target genes such as CerS6 and p21 are induced (35, 77). CerS6, which preferentially generates C_16_-ceramide, could sustain p53 stability to allow additional time for DNA repair or, if DNA is not repaired successfully, promote apoptosis. Interaction of p21 with PCNA in the nucleus inhibits replication until DNA repair is complete. In contrast, when cancer cells with defects in p53 are stressed, these mechanisms are dysregulated. Increased expression of p21 may occur in a p53-independent manner and the protein localizes to the cytoplasm where it antagonizes anti-proliferative responses induced by radiation or chemotherapy through binding with partners such as caspases or ASK1 to sustain survival. Increased expression of acid ceramidase contributes to evading cell death by hydrolyzing pro-apoptotic ceramide. Cells assume a temporary state of senescence and since p21 is localized in the cytoplasm, damaged DNA continues to replicate driving the accumulation of multiple genomes (polyploidization). Since acid ceramidase reduces ceramide levels, cholesterol metabolism is not adversely affected, and membrane fluidity remains permissive for successful budding of early progeny (depolyploidization). Although progeny formation from PGCC is estimated at only 1 in 10^5^-10^6^, successful progeny will clonally expand and give rise to subpopulations with newly acquired characteristics, including increased resistance, migration, invasion, and metastasis (10).

The goal of cancer therapy is to eliminate cancer cells but the unintentional generation of polyploid cells that withstand therapy stress and subsequently undergo depolyploidization pose a risk for recurrence. We hypothesize that preventing the formation of polyploid cancer cells during therapy stress has the potential to increase therapy efficacy and our data suggest that inhibition of p21 during therapy stress could achieve this goal. However, the only report in which the p21 inhibitor UC2288 has been used *in vivo* is a non-peer-reviewed study in a preclinical model of Parkinson’s disease (78). Although p21-deficient mice are viable, they are also more susceptible to tumor development, since p21 plays an important role in p53-dependent cell cycle arrest and tumor suppression (79). The complex nature of p21 regulation and function poses challenges for therapeutic targeting. Alternatives to preventing p21-mediated polyploidy include approaches that facilitate the killing of PGCC by adjuvant treatments with senolytics such as Navitoclax or agents that interfere with depolyploidization such as simvastatin, tamoxifen, or autophagy modulators (21, 23, 24). Thus, there may be multiple strategies to prevent depolyploidization that leads to cancer recurrence.

## Experimental procedures

### Cell culture and *in vitro* analysis

PPC1 prostate cancer was a kind gift from Dr Dean Tang (Roswell Park Comprehensive Cancer Center). U118MG and HT29 cells were obtained from Mediatech and ATCC, respectively. Identity of cell lines was verified by STTR analysis and cultures were discarded after a maximum of 30 propagations. The MycoAlert kit was used to verify cells are *mycoplasma* negative (LT07-318, Lonza). Cells were cultured in RPMI-1640 medium or DMEM (Cellgro) supplemented with 10% FBS (Hyclone) and antibiotic-antimycotic (1%; Gibco) and maintained at 37 °C with 5% CO_2_. PGCC were generated by exposing parental cells, plated 8 × 10^5^/100 mm dish overnight, to a single dose of gamma irradiation (^137^Cs γ-irradiator, J.L Sheperd & Associates), 5 nM docetaxel (Zydus Hospira Oncology) or 10 μM cisplatin (Teva Pharmaceuticals) On day 3, PGCC were captured through size exclusion filtration using a 20 micron cell strainer (PluriSelect), which results in a highly enriched PGCC working populations (21). UC2288 was obtained from Calbiochem/Sigma (#532813) and Navitoclax/ABT-263 purchased from Cell Signaling Technology (#79381). *In vitro* analysis of DNA content by flow cytometry, sphingolipids by LC/MS, and colony forming ability of PGCC was performed as previously described (24).

Pyridinium C_16_-ceramide was provided by the MUSC lipidomics shared resource/synthetic core facility (PC_16_ (D-*erythro*-2-N-[6′-(1′′-pyridinium)-hexanoyl]-sphingosine bromide) (http://www.hollingscancercenter.org/research/shared-resources/lipidomics/index.html). Replication-deficient adenoviral vectors (Ad5) were obtained from Vector Biolabs (AdLuc, #1000) or Dr Sunil Chada (Adp53, (33, 80)). PPC1 cells were plated at 8 × 10^5^ cells/100 mm dish overnight, and transduced with 1 MOI of viral vector for 24 h before adding 5 μM pyridinium C_16_-ceramide for an additional 24 h.

### RNA-seq analysis

PPC1 cells were plated at 7 × 10^5^ on 100 mm plates on day 1. On day 2, three sets of plates were exposed to 8 Gy radiation. On day 4, the untreated control populations and one irradiated set were washed and trypsinized. To collect PGCC, cells from the irradiated set were passed through a 20-micron cell strainer (Pluriselect), and the captured cells eluted. Early and late progeny were collected on days 8 and 20, respectively as above, except that the flowthrough was collected. The experiment was performed with 6 replicates, for a total of 24 samples. RNA was isolated (Qiagen RNAEasy kit) and submitted to Novogene for processing and basic analysis of raw data. Workflows for generation of the library and subsequent bioinformatic analysis have been published in detail as supplementary material (21). Briefly, prior to library construction, RNA integrity was verified using the Agilent 2000. Downstream analysis was performed using a combination of programs including STAR, HTseq, Cufflink and wrapped scripts. Alignments were parsed using the Tophat program and differential expressions were determined through DESeq2. GO and KEGG enrichment were implemented by the ClusterProfiler (81, 82, 83, 84, 85, 86, 87, 88, 89).

### Bioinformatic analysis

Additional bioinformatic analysis was performed using the Network Analyst database. Comparisons were made between untreated/PGCC, PGCC/early progeny, early/late progeny, untreated/early progeny, and untreated vs. late progeny. To construct the gene expression networks of giant, early, and late progeny cell states, genes differentially expressed (*p-adj* = 0.001 and log2FC = 1.5/−1.5) in untreated *versus* giant, untreated *versus* early and untreated *versus* late progeny states are uploaded to the Network Analyst Database (90, 91). The nodes in each network are sized and color coded based on their degree and betweenness, which correspond to the number of connections a node has, and the number of paths that pass through it when considering the pairwise shortest paths between all nodes in the network, respectively. To analyze the effects of the genes associated with different cell states on clinical outcome (1), a PGCC score comprising the differentially expressed genes in untreated *versus* giant groups or (2) a 5-genes signature for the giant and late progeny states comprising 5 central hubs that have the highest degree and betweenness were constructed. The Kaplan Meier survival analysis for cervical squamous cell carcinoma, endocervical adenocarcinoma, stomach adenocarcinoma, kidney renal papillary cell carcinoma, low-grade glioma, uveal melanoma, mesothelioma, lung squamous cell carcinoma, sarcoma and prostate adenocarcinoma was performed using the TCGA dataset (https://www.cancer.gov/tcga) *via* utilizing the GEPIA2 (92). The prostate cancer dataset GSE116918 that contains gene expression data from 248 patients with localized/locally advanced prostate cancer undertaking radical radiotherapy (with androgen deprivation therapy) was downloaded from the GEO database, https://www.ncbi.nlm.nih.gov/geo/query/acc.cgi (93). For the survival analysis using the GEPIA2 database, only the upregulated genes in the PGCC score were uploaded into the database to generate the PGCC score. The scores of the PGCC signature for the GSE116918 dataset were calculated by subtracting the z-scores of the genes downregulated from the z-scores of the genes upregulated in the signature using the SPSS Statistics software as described previously (94). The 5-genes signature scores were calculated by summing up the z-scores of the 5 genes in the signature using the SPSS Statistics software. Survival curves were generated based on median or 25th percentile separation using Kaplan-Meier method, and significance between groups was calculated by Log-rank test. For correlation analysis, Pearson correlation coefficients were calculated.

#### Western blotting

Cell pellets were processed in RIPA to obtain whole cell extracts. For fractionation, pellets were lysed in 10 mM HEPES, 1.5 mM MgCl, 10 mM KCl, 0.5 mM DTT, 0.05% NP40, pH7.9 with freshly added proteinase inhibitors and centrifuged at 3000 rpm at 4 ^°^C for 10 min. The supernatant was collected as the cytosolic fraction. The pellet was washed and resuspended in 5 mM HEPES, 1.5 mM MgCl, 0.2 mM EDTA, 0.5 mM DTT, 26% glycerol (v/v), pH 7.9. NaCl was added to a final concentration of 300 mM and material was lysed by 20 strokes with a Dounce homogenizer. After 30 min on ice, samples were centrifuged at 24,000*g* for 20 min at 4 ^°^C. Western blotting was performed as previously described (95). Primary antibodies were p21 (1:1000, Cell Signaling cat# 2947), p53 (1:1000, Cell Signaling cat# 9282), acid ceramidase (1:1000 cat# 612302, BD Transduction Laboratories), Histone H3 (1:1000, Cell Signaling, cat#4499), actin (1:2000, cat# A2066, Sigma) or GAPDH (1:4000 cat# sc32233, Santa Cruz, Dallas, TX). Secondary antibodies were obtained from Santa Cruz Biotechnology (anti-mouse cat# sc-2005, 1:20,000 for GAPDH or 1:5000 for acid ceramidase) and anti-rabbit cat# sc-2004, 1:5000). The Immobilon Western Chemiluminescent HPR substrate (WBKLS0100, Millipore) was used to detect signals either on film or by Chemi-Doc Imager (BioRad). Signals were quantified with ImageJ software. Statistical analysis was performed with GraphPad software.

## Data availability

All data generated and analyzed during the current study are included in this published article (and Supplementary Information files). The datasets generated and analyzed during the current study are available in the Gene Expression Omnibus (GEO) database repository accession GSE196453 https://www.ncbi.nlm.nih.gov/geo/query/acc.cgi?acc=GSE196453.

## Supporting information

This article contains supporting information.

## Conflict of interest

The authors declare that they have no conflicts of interest with the contents of this article.
