# Supplementary material for: Transcriptome analysis of polyploid giant cancer cells and their progeny reveals a functional role for p21 in polyploidization and depolyploidization
Source: J Biol Chem. 2024 Mar 4;300(4):107136. doi: 10.1016/j.jbc.2024.107136 (PMC10979113; doi:10.1016/j.jbc.2024.107136)
Supplement: Supporting Figures S1–S4 and Tables S1 and S2 [file mmc1.pdf]

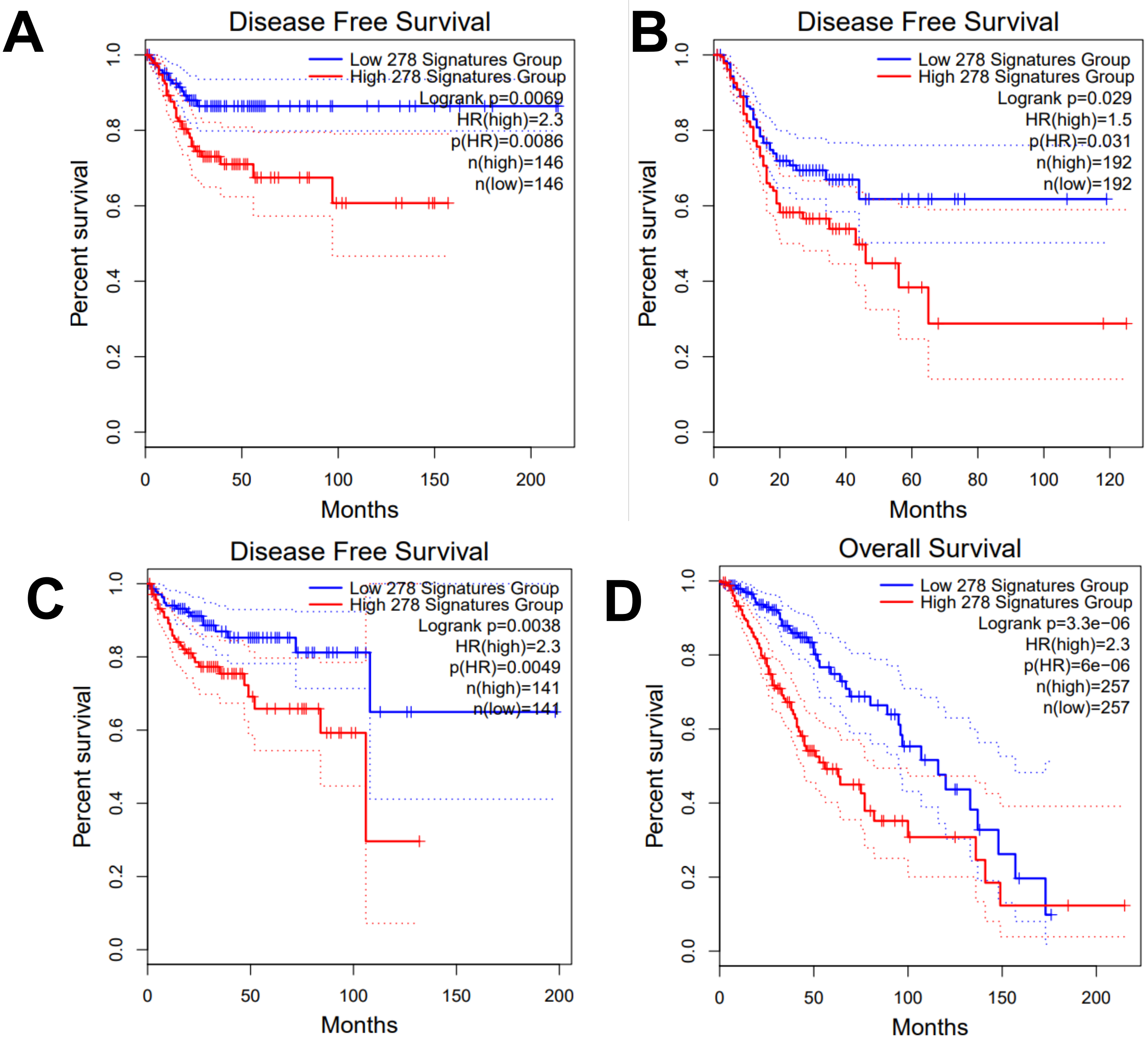

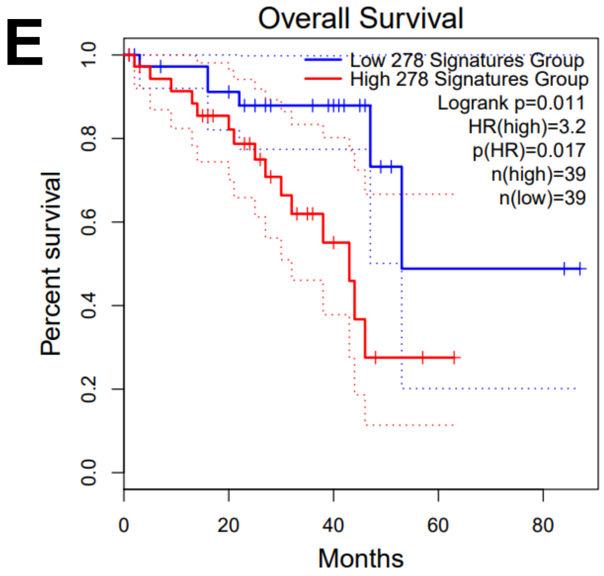

Figure S1. The PGCC signature predicts survival in multiple malignancies. (A) Cervical squamous cell carcinoma and endocervical adenocarcinoma (p=0.0086). (B) Stomach adenocarcinoma (p=0.031). (C) Kidney renal papillary cell carcinoma (p=0.0049). (D) Low grade glioma ($p=6x10^{-6}$). (E) Uveal melanoma (p=0.017).

A

GeneName	padj(UTvsGiant)
CDKN1A	1.27E-77
CDKN2A	0.36596
GLB1	2.59E-19
IL-1B	1.00E-83
IL-6	NA
IL-8	5.34E-06
TP53	6.16E-15

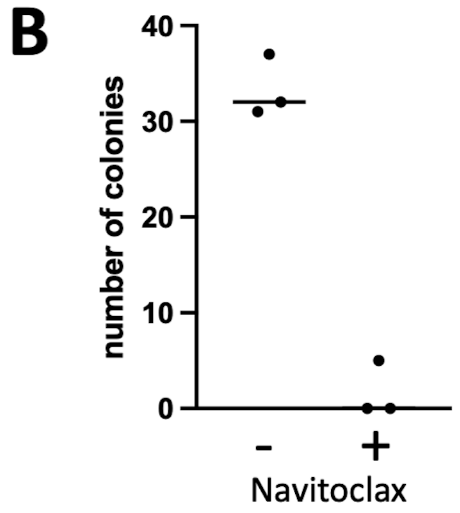

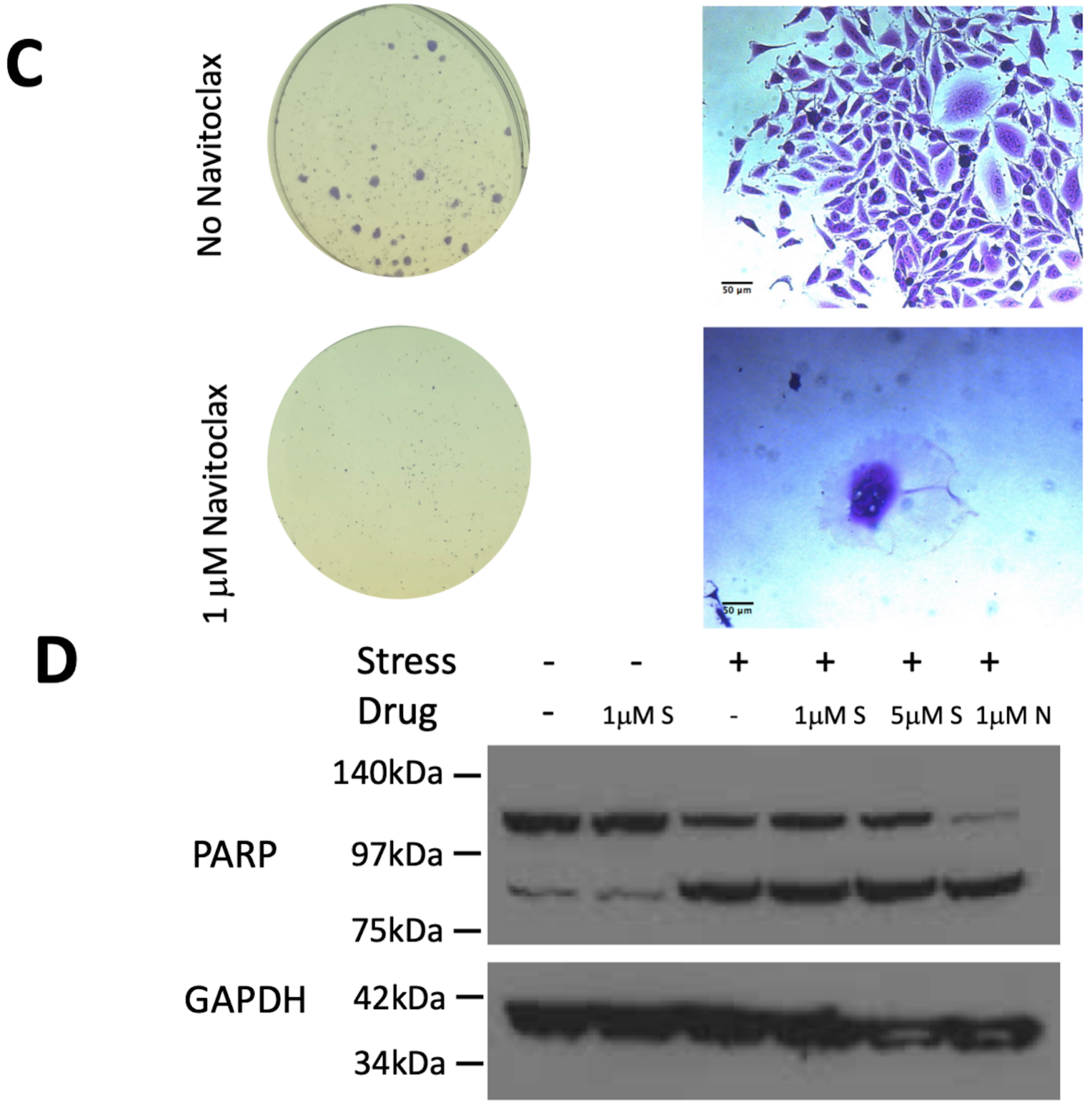

Figure S2. PGCC and senescence in PPC1 cells. (A) Expression of senescence genes in PGCC vs. untreated cells. (B) Colony formation of PGCC in the absence and presence of 1μM Navitoclax, n=3. (C) Representative images of colony formation. Scale bar in right panels is 50 μm (D). Western blot analysis of PARP cleavage in PGCC treated with 1 or 5 μM Simvastatin or 1μM Navitoclax. Navitoclax but not simvastatin enhanced PARP cleavage in PGCC.

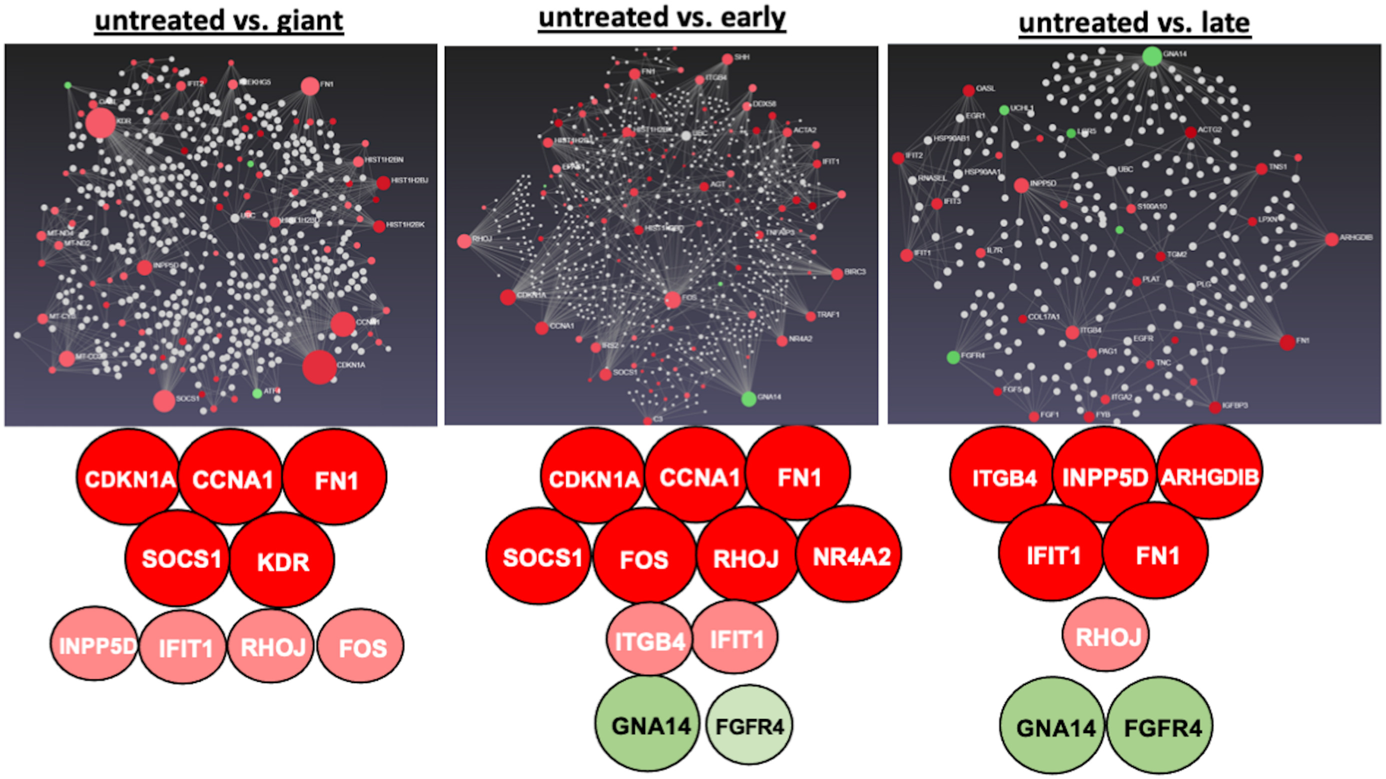

Figure S3. Identification of central hubs in PGCC and progeny cells. The untreated vs. PGCC, untreated vs. early progeny and untreated vs. late comparisons were used to determine the most important elements of each cell state. Genes that are shown in red are upregulated in the corresponding group as compared to untreated throughout the transformation from PGCC to early to late progeny. IFIT1 is upregulated in PGCC but it is not among the central hubs in the network becoming only a central hub in late progeny. Among the down genes, GNA14, which is downregulated in early and late progeny, is also among the central hubs in these populations. FGFR4 is downregulated in early progeny but only becomes the central hub in the late progeny. Note that the panel on the top left was reused as Figure 2A in the main manuscript.

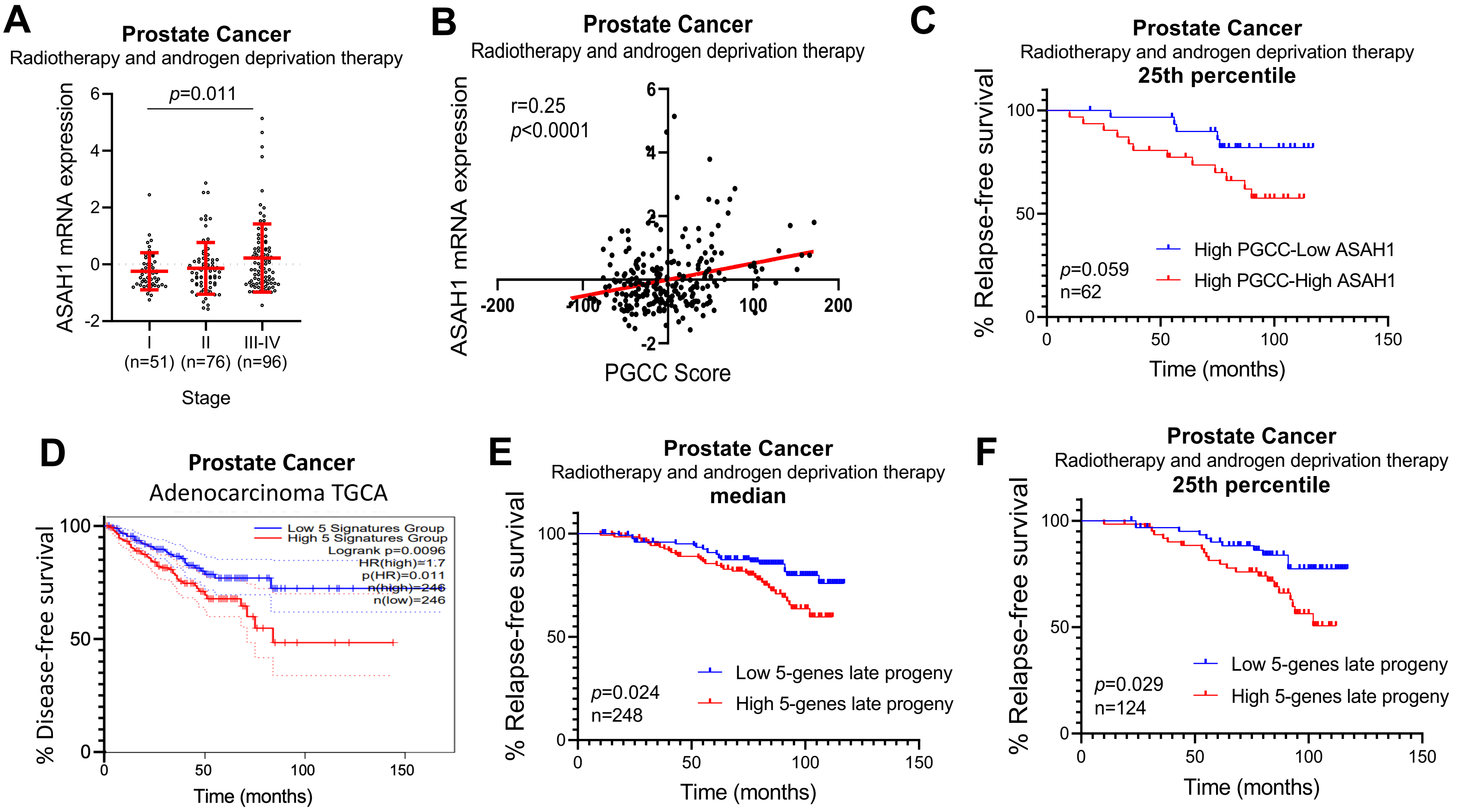

Figure S4. Analysis of expression profiles in prostate cancer. (A-C) Data from GSE116918. (A) ASAH1 expression is higher in high stage tumors of prostate cancer patients treated with radiotherapy and androgen deprivation therapy (B) PGCC score correlates with ASAH1 expression. (C) Among patients with high PGCC score (separated from median), high ASAH1 expression (separated from 25th percentile) trends towards predicting worse relapse-free survival. (D-F) Late progeny core upregulated signature (FN1, INPP5D, ITGB4, ARHGDIB and IFIT1) significantly predicts disease-free survival in TGCA prostate adenocarcoma patients (D) and relapse-free survival in GSE116918 patients (E, median; F, 25th percentile separation).

GeneName	log2Fold	p-value	GeneDescription
MMP24	1.70	1.207E-05	matrix_metallopeptidase_24_(membrane-inserted)
IZUMO4	1.53	1.4216E-05	IZUMO_family_member_4
CPA4	4.19	2.4477E-05	carboxypeptidase_A4
TRIM54	1.70	2.6875E-05	tripartite_motif_containing_54
PLAT	3.51	3.4723E-05	plasminogen_activator_tissue
CAND2	1.87	5.8755E-05	cullin-associated_and_neddylation-dissociated_2_(putative)
GPR56	2.22	0.00013287	G_protein-coupled_receptor_56
INHBA	2.59	0.00023523	inhibin_beta_A
IL1B	1.64	0.00035248	interleukin_1_beta
PRSS23	1.60	0.00035639	protease_serine_23
HHIPL2	4.00	0.00046635	HHIP-like_2
FN1	1.60	0.00118225	fibronectin_1
IFIT2	1.94	0.00147863	interferon-induced_protein_with_tetratricopeptide_repeats_2
P2RY6	3.67	0.00240607	pyrimidinergic_receptor_P2Y_G-protein_coupled_6
TNFRSF9	2.23	0.00264261	tumor_necrosis_factor_receptor_superfamily_member_9
DNM3	1.89	0.00366889	dynamin_3
FLRT1	2.75	0.00441904	fibronectin_leucine_rich_transmembrane_protein_1
EGF	1.84	0.00451331	epidermal_growth_factor
CFH	3.97	0.00535219	complement_factor_H
AGT	1.81	0.00540075	angiotensinogen_(serpin_peptidase_inhibitor_clade_A_member_8)
TUBB2B	1.66	0.0058183	tubulin_beta_2B_class_IIb
CALHM3	2.70	0.00671915	calcium_homeostasis_modulator_3
MB	1.87	0.00672373	myoglobin
SEMA7A	1.59	0.00899396	semaphorin_7A_GPI_membrane_anchor_(John_Milton_Hagen_blood_group)
GRIN2D	2.35	0.00902554	glutamate_receptor_ionotropic_N-methyl_D-aspartate_2D
CCL26	2.18	0.00912581	chemokine_(C-C_motif)_ligand_26
C11orf96	2.19	0.01311367	chromosome_11_open_reading_frame_96
FBXW10	4.24	0.01769589	F-box_and_WD_repeat_domain_containing_10
SDPR	2.18	0.02293982	serum_deprivation_response
IFITM1	2.19	0.04015274	interferon_induced_transmembrane_protein_1
DKK3	3.07	0.04105634	dickkopf_3_homolog_(Xenopus_laevis)

Supplemental Table S1. Overlap of radiation resistance genes. We analyzed the overlap of genes that are upregulated in radiation resistant Du145 cells compared to controls (GSE53902, 1.5-fold) and PPC1 PGCC. Among the 781 genes in the Du145 dataset, 554 were identified in PGCC. Of the 554 genes, 31 were increased by at least 2-fold in PGCC and significantly different from untreated controls.

GeneName	log2Fold	p-value	GeneDescription
BMX	3.94	0.00291028	BMX_non-receptor_tyrosine_kinase
C1orf116	1.82	0.00039658	chromosome_1_open_reading_frame_116
CCL26	2.18	0.00912581	chemokine_(C-C_motif)_ligand_26
CHD5	1.80	0.00798422	chromodomain_helicase_DNA_binding_protein_5
DAPP1	4.56	0.03051498	dual_adaptor_of_phosphotyrosine_and_3-phosphoinositides
GPR87	2.62	0.0016323	G_protein-coupled_receptor_87
NFAM1	1.79	0.02305345	NFAT_activating_protein_with_ITAM_motif_1
PROX2	1.67	0.04592944	prospero_homeobox_2
RNF182	2.11	0.00057721	ring_finger_protein_182
SIGIRR	1.77	0.00430471	single_immunoglobulin_and_toll-interleukin_1_receptor_(TIR)_domain
SLC39A12	3.19	0.00066199	solute_carrier_family_39_(zinc_transporter)_member_12
SUSD2	2.24	0.04717655	sushi_domain_containing_2
TNFRSF25	2.14	0.00110427	tumor_necrosis_factor_receptor_superfamily_member_25
TNS4	1.57	0.00516216	tensin_4
VNN1	2.57	0.00010407	vanin_1
WNT9A	1.51	0.00025584	wingless-type_MMTV_integration_site_family_member_9A

Supplemental Table S2. Overlap of stemness genes. We analyzed the overlap of genes that were upregulated in $ALDH^+$ DU145 cells compared to $ALDH^-$ cells (GSE53902, 1.5-fold) and PPC1 PGCC. Among the 428 genes in the Du145 dataset, 242 were identified in PGCC. Of the 242 genes, 16 were increased by at least 2-fold in PGCC and significantly different from untreated controls.
